# Viral metagenomic analysis of feces of wild small carnivores

**DOI:** 10.1186/1743-422X-11-89

**Published:** 2014-05-15

**Authors:** Rogier Bodewes, Aritz Ruiz-Gonzalez, Claudia ME Schapendonk, Judith MA van den Brand, Albert DME Osterhaus, Saskia L Smits

**Affiliations:** 1 Department of Viroscience, Erasmus Medical Center, Rotterdam, The Netherlands; 2 Department of Zoology and Animal Cell Biology, University of the Basque Country (UPV/EHU), Vitoria-Gasteiz, Spain; 3 Systematics, Biogeography and Population Dynamics Research Group, Lascaray Research Center, University of the Basque Country (UPV/EHU), Vitoria-Gasteiz, Spain; 4 Conservation Genetics Laboratory, National Institute for Environmental Protection and Research (ISPRA), Bologna, Ozzano dell’Emilia (BO), Italy; 5 ViroClinics BioSciences BV, Rotterdam, The Netherlands

**Keywords:** Theilovirus, Viral metagenomics, Picobirnavirus, Amdovirus, Phlebovirus, Carnivore, Wild carnivore, Wildlife, Red fox, Fox, Otter, Common gennet, Bunyavirus

## Abstract

**Background:**

Recent studies have clearly demonstrated the enormous virus diversity that exists among wild animals. This exemplifies the required expansion of our knowledge of the virus diversity present in wildlife, as well as the potential transmission of these viruses to domestic animals or humans.

**Methods:**

In the present study we evaluated the viral diversity of fecal samples (n = 42) collected from 10 different species of wild small carnivores inhabiting the northern part of Spain using random PCR in combination with next-generation sequencing. Samples were collected from American mink (*Neovison vison*), European mink (*Mustela lutreola*), European polecat (*Mustela putorius*), European pine marten (*Martes martes*), stone marten (*Martes foina*), Eurasian otter (*Lutra lutra*) and Eurasian badger (*Meles meles*) of the family of Mustelidae; common genet (*Genetta genetta*) of the family of Viverridae; red fox (*Vulpes vulpes*) of the family of Canidae and European wild cat (*Felis silvestris*) of the family of Felidae.

**Results:**

A number of sequences of possible novel viruses or virus variants were detected, including a theilovirus, phleboviruses, an amdovirus, a kobuvirus and picobirnaviruses.

**Conclusions:**

Using random PCR in combination with next generation sequencing, sequences of various novel viruses or virus variants were detected in fecal samples collected from Spanish carnivores. Detected novel viruses highlight the viral diversity that is present in fecal material of wild carnivores.

## Background

Transmission of viruses from wildlife to humans continues to cause outbreaks of disease in humans. Examples of recent outbreaks are the Middle East Respiratory Syndrome-coronavirus (MERS-CoV) that may have originated from bats and/or camelids and the influenza A (H7N9) virus that originated from wild birds [1-5]. A systematic exploration of viruses present in several key host species of wild animals might provide important information to find the original host or carriers of viruses of future outbreaks of viral disease among domestic animals, endangered animal species, and humans [6]. Furthermore, information about the presence of viruses in healthy hosts provides a baseline level for viruses present in these animals in case an outbreak of disease occurs. In previous viral metagenomics studies, high numbers of new viruses have been identified [7-10]. The results of these studies have highlighted that our knowledge of the viral reservoir is far from complete and many, as yet, unidentified viruses circulate among humans and wild and domestic animals. However, there is an enormous diversity of viral sequences and viral metagenomics efforts should be focused on outbreaks of disease and viral metagenomics on samples collected from a selected number of key species [11].

Wild carnivores are known carriers of several viral pathogens that can affect domestic animals and humans, including rabies and canine distemper virus [12,13]. In addition, in previous studies various previously unknown viruses have been detected in European badgers, red foxes and European pine martens in the Netherlands [9,14]. In the present study, we evaluated the viral diversity of fecal swabs or fecal specimens collected from 10 different small carnivore species of the Mustelidae, Canidae, Viverridae and Felidae families inhabiting northern Spain.

## Results and discussion

### Metagenomic overview

**Figure 1:**
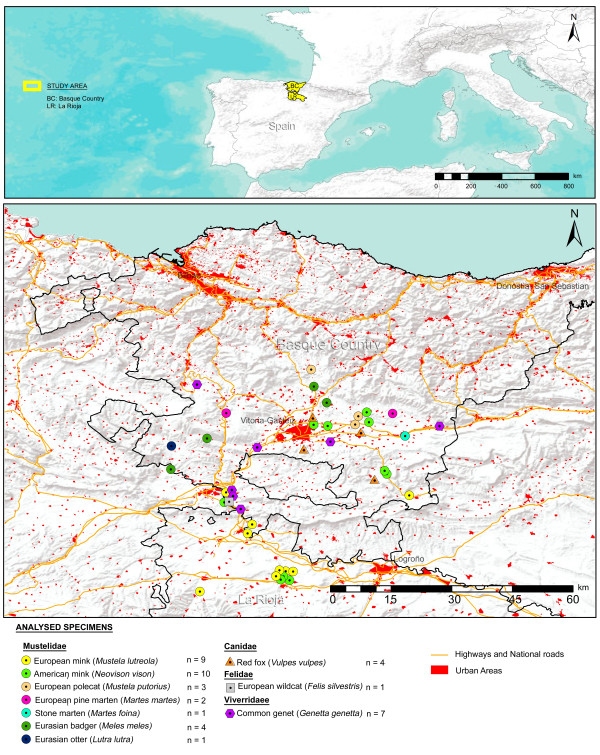
**Map with locations of sample collection.** Maps showing the location of the study area in the Basque Country and La Rioja regions (Spain) and spatial distribution of the 42 analysed carnivore samples of the Mustelidae, Canidae, Felidae and Viverridae families.

**Figure 2:**
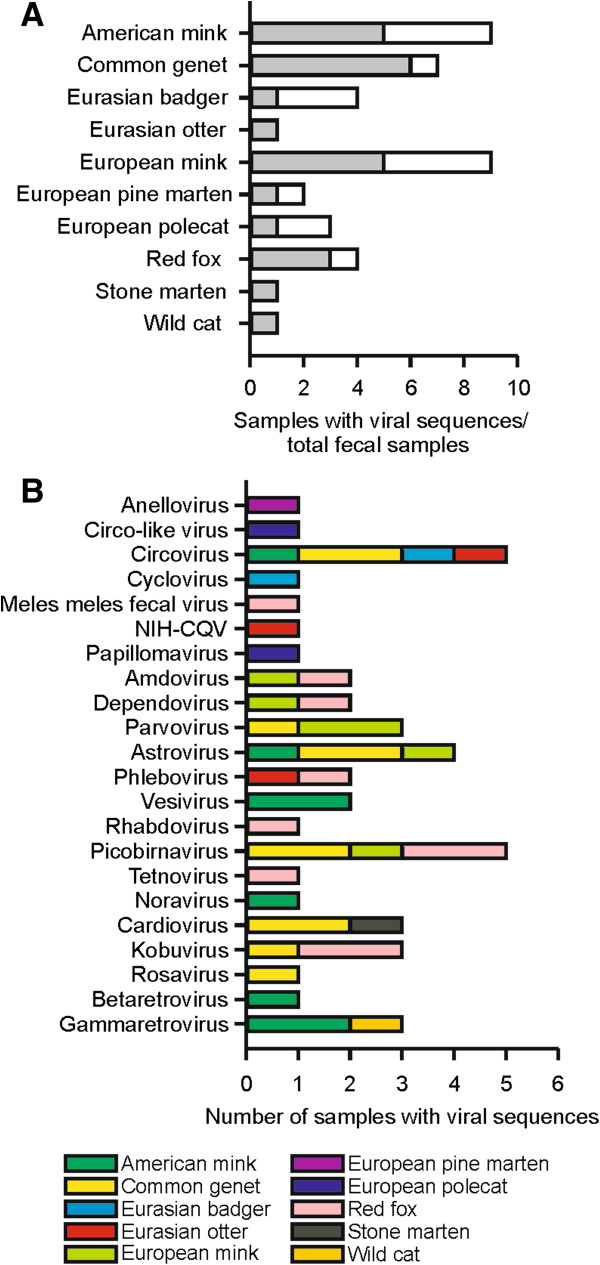
**Overview of detected viral sequences in the present study.** Number of animals included in this study (bars) and the number of samples in which viruses where detected (grey areas; **A)**. Number of animals in which viral sequences were detected for each genus/ group **(B)**.

Using random amplification in combination with next-generation sequencing, more than 320,000 trimmed sequence reads were obtained of fecal samples collected from the carnivores of the present study (Figure 1). Reads were classified into eukaryotic viruses, phages, bacteria and eukaryotes. Many of the identified sequences were of bacterial or eukaryotic origin. A substantial proportion of the reads did not have any significant hits for nucleotide or amino acid sequences in GenBank. In addition, several reads were detected that had the closest similarity to viruses. In the majority of the samples, sequences of the order *Caudovirales* were detected and in 26 out of 42 samples, sequences were detected that had the closest similarity to viruses known to infect eukaryotes (Figure 2A, Table 1). Viruses belonging to the families of *Anelloviridae*, *Astroviridae*, *Bunyaviridae*, *Caliciviridae*, *Circoviridiae*, *Parvoviridae* subfamily *Parvovirinae*, *Picobirnaviridae*, *Picornaviridae*, *Rhabdoviridae*, and *Retroviridae* were detected (Figure 2B). Furthermore, sequences were detected that had the closest similarity to the recently proposed family of *Breviviridae* and the recently described hybrid DNA virus NIH-CQV/PHV which was identified as a contaminant of silica column-based nucleic acid extraction kits [9,15,16]. No sequences were detected that were identical to currently known zoonotic viruses. A proportion of the detected viral sequences had the closest similarity to viruses previously detected in birds and rodents. For example, in an European mink (sample 26), sequences were detected with >95% homology on the nucleotide level with Turkey parvovirus and in a stone marten (sample 41), sequences were detected with 94-96% homology on the nucleotide level with Encephalomyocarditis virus type 2 isolate RD 1338 (D28/05) detected in a wood mouse (*Apodemus sylvaticus*) [17]. These viruses most likely originate from the diet of the animals. In addition, sequences with >95% identity on the nucleotide level to viruses that are known to infect mink were detected in European and American mink, including Mink calicivirus strain MCV-DL/2007/CN [18] (samples 1 and 8) and Aleutian mink disease virus (sample 30). Antibodies to Aleutian mink disease parvovirus have been detected in a cohort of free-ranging European mink in southwestern France and northern Spain previously, but not in another cohort of free-ranging European mink in Navarra, Spain [19-21]. Additional sampling and confirmation by specific PCR is necessary to indeed confirm that the Aleutian mink disease parvovirus is circulating among these animals. Besides these sequences that had high homology with known viruses, also sequences were detected that had the closest similarity to viruses, but with only low homology. A number of sequences of potentially novel viruses or virus variants, including a theilovirus, phleboviruses, an amdovirus, a kobuvirus and picobirnaviruses, were further characterized in the present manuscript, while sequences of the other viruses are preliminary and need further characterization.

**Table 1:** Overview of samples used in this study and detected mammalian viral sequences

**No**	**Species**	**Date of collection (dd/mm/yyyy)**	**Observations**^**1**^	**Analyzed**^**2**^	**Reads**	**Detected viral sequences**^**3,4**^
1	American mink (*Neovison vison*)	08/11/2012	e	F	5316	t.b.c.
2	American mink (*Neovison vison*)	15/11/2012	e	RS	4168	t.b.c.
3	American mink (*Neovison vison*)	10/11/2012	e	F	7563	t.b.c.
4	American mink (*Neovison vison*)	09/11/2012	e	F	9041	n.d.
5	American mink (*Neovison vison*)	19/09/2012	e	F	4919	t.b.c.
6	American mink (*Neovison vison*)	13/10/2012	e	F	4454	n.d.
7	American mink (*Neovison vison*)	21/09/2012	e	F	4587	n.d.
8	American mink (*Neovison vison*)	26/10/2012	e	F	5592	t.b.c.
9	American mink (*Neovison vison*)	11/09/2012	e	F	5838	n.d.
10	American mink (*Neovison vison*)	10/04/2012	e	F	6925	n.d.
11	Common genet (*Genetta genetta*)	08/11/2012	lt	RS	6353	t.b.c.
12	Common genet (*Genetta genetta*)	05/11/2012	fd	F	8642	t.b.c.
13	Common genet (*Genetta genetta*)	04/07/2012	lt	RS	5582	t.b.c.
14	Common genet (*Genetta genetta*)	09/11/2012	fd	F	5217	Cardiovirus, Picobirnavirus, t.b.c.
15	Common genet (*Genetta genetta*)	14/12/2012	fd	RS	6758	n.d.
16	Common genet (*Genetta genetta*)	19/02/2013	fd	RS	7327	t.b.c.
17	Common genet (*Genetta genetta*)	10/05/2013	fd	RS	9068	t.b.c.
18	Eurasian badger (*Meles Meles*)	04/01/2013	fd	RS	6465	t.b.c.
19	Eurasian badger (*Meles Meles*)	13/05/2013	fd	RS	11228	n.d.
20	Eurasian badger (*Meles Meles*)	12/05/2013	fd	RS	3763	n.d.
21	Eurasian badger (*Meles Meles*)	21/05/2013	fd	RS	6946	n.d.
22	Eurasian otter (*Lutra lutra*)	09/01/2013	fd	F	5500	Phlebovirus, t.b.c.
23	European mink (*Mustela lutreola*)	06/11/2012	lt	RS	11356	t.b.c.
24	European mink (*Mustela lutreola*)	07/12/2012	lt	RS	13933	t.b.c.
25	European mink (*Mustela lutreola*)	08/11/2012	lt	RS	13119	n.d.
26	European mink (*Mustela lutreola*)	16/11/2012	lt	RS	7242	t.b.c.
27	European mink (*Mustela lutreola*)	26/11/2012	fd	RS	10046	t.b.c.
28	European mink (*Mustela lutreola*)	7/03/2013	lt	RS	9027	n.d.
29	European mink (*Mustela lutreola*)	8/03/2013	lt	RS	7391	n.d.
30	European mink (*Mustela lutreola*)	6/03/2013	lt	RS	6593	t.b.c.
31	European mink (*Mustela lutreola*)	20/03/2012	fd	RS	9418	n.d.
32	European pine marten (*Martes martes*)	11/11/2012	fd	F	9624	n.d.
33	European pine marten (*Martes martes*)	05/03/2013	fd	RS	9961	t.b.c.
34	European polecat (*Mustela putorius*)	19/02/2013	lt	RS	7422	t.b.c.
35	European polecat (*Mustela putorius*)	28/02/2013	fd	RS	9741	n.d.
36	European polecat (*Mustela putorius*)	15/05/2013	fd	RS	17970	n.d.
37	Red fox (*Vulpes vulpes*)	08/01/2013	fd	RS	5163	Kobuvirus, Phlebovirus
38	Red fox (*Vulpes vulpes*)	29/01/2013	fd	RS	5722	n.d.
39	Red fox (*Vulpes vulpes*)	17/03/2013	fd	F	5842	Kobuvirus, t.b.c.
40	Red fox (*Vulpes vulpes*)	08/04/2013	fd	F	8828	Picobirnavirus, Amdovirus, t.b.c.
41	Stone marten (*Martes foina*)	15/11/2012	fd	F	6809	t.b.c.
42	Wild cat (*Felis silvestris*)	24/05/2013	fd	RS	7224	t.b.c.

^1^e: euthanized animal (in the framework of an eradication program of invasive species), lt: live trapped animal, fd: found dead.

^2^F: fecal sample, R: rectal swab.

^3^nd: no viral sequences detected.

^4^t.b.c.: one or more sequences were detected that were most closely related to viral sequences, but sequences were not characterized in the present manuscript.

### Genet fecal theilovirus

Picornaviruses are small, positive-stranded non-enveloped RNA viruses. This large family was subdivided into several genera [22]. The genus *Cardiovirus* of the family *Picornaviridae* currently consists of two species, Theilovirus and Encephalomyocarditis virus (EMCV) [22]. Viruses belonging to the species Theilovirus were detected initially mainly in rodents. In these animals, viruses cause primarily infection of the digestive tract without clinical signs, but extra-intestinal infection occurs and can cause an acute encephalomyelitis and a chronic demyelinating infection of the central nervous system [23]. Vilyuisk human encephalomyelitis virus, another strain of the species Theilovirus, was isolated from humans with encephalomyelitis, but only after serial passage over mice brains [24,25]. More recently, Saffold virus was discovered in a stool sample of a child with fever of unknown origin, and additional research revealed that infection with this virus was common [26-28].

**Figure 3:**
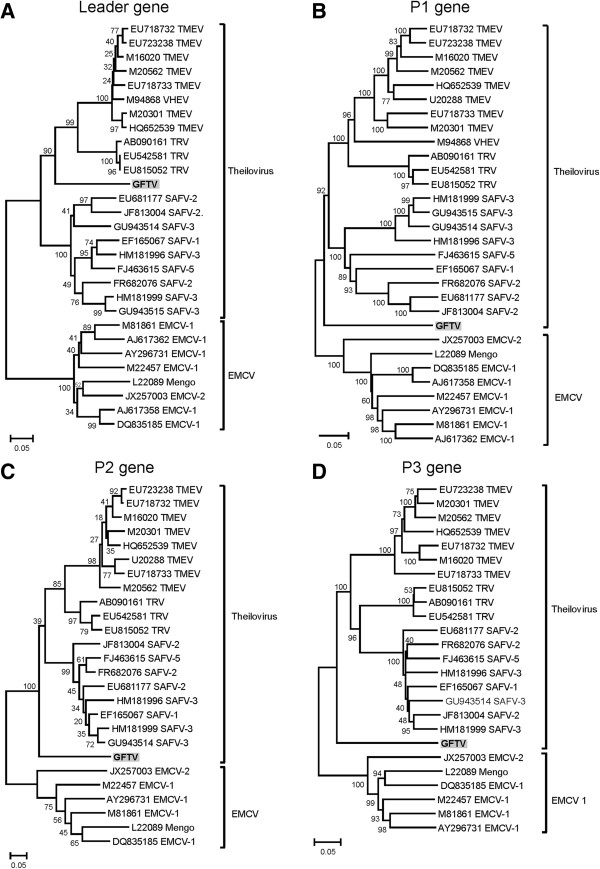
**Phylogenetic analysis of Genet fecal theilovirus (GFTV).** Phylogenetic neighbor-joining tree with *p*-distance and 1,000 bootstrap replicates of the nucleotide sequence of the Leader gene **(A)**, P1 gene **(B)**, P2 gene **(C)** and partial P3 gene **(D)** of Genet fecal theiloviruses and various other viruses of the genus *Cardiovirus*. TMEV: Theiler’s murine ecephalomyelitis vrus, VHEV: Vilyuisk human encephalomyelitis virus, TRV: Theiler’s-like rat virus, SAFV: Saffold virus, EMCV: encephalomyocarditis virus. Indicated are genbank accession numbers and bootstrap values.

We detected in fecal material of a stone marten (sample 41) sequences with high similarity to a novel Encephalomyocarditis type 2 virus detected recently in a wood mouse (*Apodemus sylvaticus*) [17]. In addition, sequences were detected that had the closest similarity to viruses of the species Theilovirus in a rectal swab collected from a common genet (sample 14). The obtained sequences were further analyzed by designing primers based on the obtained sequence reads and the partial sequence (6488 nt) of a novel theilovirus, tentatively called Genet fecal theilovirus (GFTV; Genbank accession KF823815), was obtained. Despite various attempts using degenerate primers and 3’ RACE PCRs, no more sequences could be obtained of the 5’ and 3’ end, possibly due to low virus genome copy numbers present in the original material. The obtained sequence contained the partial non-coding region of the 5’end and the partial ORF encoding for the polyprotein gene. Based on splice-site predictions and alignment with other theiloviruses, the complete coding sequence of the L protein (228 nt), the complete P1 (2462 nt) capsid-encoding regions, the complete P2 (1785 nt) non-structural coding region and the partial P3 (1455 nt) non-structural coding region were detected in the partial polyprotein. Pairwise identity analysis and phylogenetic analysis of the nucleotide sequences of the complete Leader gene, P1, P2 and partial P3 gene showed that Genet fecal theilovirus probably belongs to a novel genotype, with maximum pairwise identities on the nucleotide (and deduced amino acid) level of respectively 69 (70), 63 (68), 62 (65) and 82 (65)% (Figure 3). Additional alignment of the deduced amino acid sequence of the P1 gene with the major surface structures of other viruses of the species theilovirus (VP2 puffs A and B, the VP3 knob and VP1 loops 1 and 2) indeed shows the high divergence between the potential immunogenic sites of these viruses (Additional file 1: Figure S1) [29,30]. In addition to the major ORF encoding the polyprotein, an alternative ORF of 423 nt (140AA) was present in the Genet fecal theilovirus sequence based on an alternative initiation codon 13 nt downstream of the authentic initiation codon, which indicates that the L* protein previously observed in Theiler’s murine encephalomyocarditis virus and other strains of the species Theilovirus is also present in this novel virus [29,31]. Since this animal was found dead, possibly due to a car accident, and the carcass was stored at −20 for a few months, a necropsy could be performed. The carcass was defrosted but no abnormalities were detected in this animal upon macroscopic and microscopic examination of various tissues, including the brain. This indicates that the novel Genet fecal theilovirus has not caused any significant disease. Since picornaviruses are known to be very resistant to a low pH, further research needs to be performed to elucidate whether the detected virus is derived from the prey species of this animal or is a novel theilovirus of the common genet.

### Novel phleboviruses in feces of a red fox and an eurasian otter

Bunyaviruses are segmented, negative-sense single stranded RNA viruses. Genomes consist of three different segments, the Large (L), Medium (M) and Small (S) segment. At present the ICTV has recognized five different genera, *Orthobunyavirus*, *Hantavirus*, *Nairovirus*, *Phlebovirus*, and *Tospovirus*[22]. The genus *Phlebovirus* consists of a genetically diverse group of viruses, some of which were described very recently [32-34]. A few members of this genus were identified as important pathogens in humans and domestic animals, including Rift valley fever virus and the Severe fever with thrombocytopenia syndrome virus or Huaiyangshan virus [35,36].

**Figure 4:**
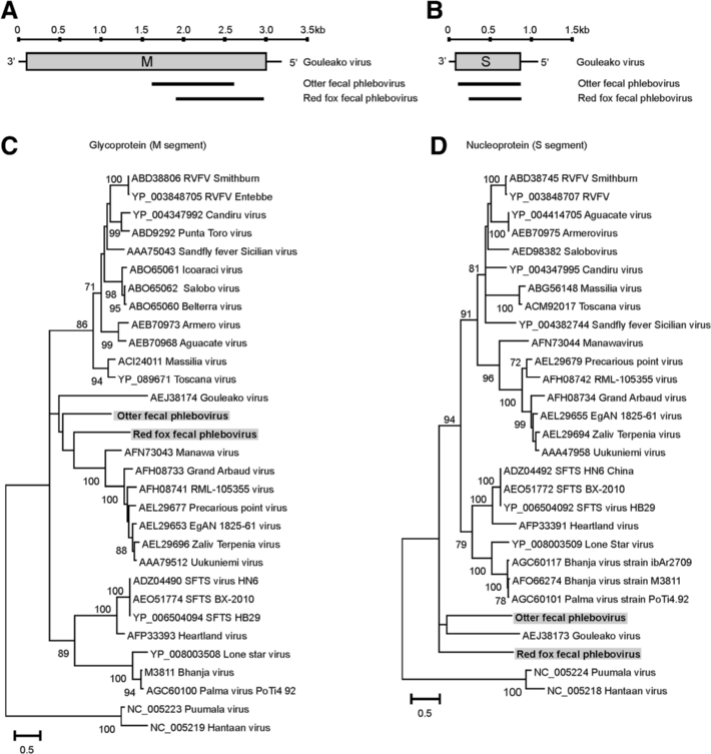
**Phylogenetic analysis of Red fox fecal phlebovirus and Otter fecal phlebovirus.** Overview of obtained viral sequence of the M segment **(A)** and S segment **(B)** of Otter fecal phlebovirus and Red fox fecal phlebovirus using Gouleako virus as a reference. Phylogenetic maximum likelihood tree (WAG + F + I + G model) with 100 bootstrap replicates of the deduced amino acid sequence of the partial glycoprotein gene **(C)** and nucleoprotein gene **(D)** of Red fox and Otter fecal phleboviruses and various other viruses of the genus *Phlebovirus*. RVFV: Rift valley fever virus, SFTS: Severe fever with thrombocytopenia syndrome virus. Indicated are Genbank accession numbers. Only bootstrap values above 70 are indicated.

In fecal material of an Eurasian otter (sample 22) and a red fox (sample 37) sequences were detected that had the closest similarity to viruses of the genus *Phlebovirus* (Table 1). Additional virus reads were obtained of the samples of these animals (total 43,584 of sample 22 and 30,064 reads of sample 37). By analysis of the additional obtained sequences of sample 22, the partial viral nucleoprotein (NP) gene (S segment) and partial glycoprotein (G) gene (M segment) of a novel phlebovirus, tentatively called Otter fecal phlebovirus (NP gene 699 nt, covered by 16 reads, G gene 984 nt, covered by 59 reads, Genbank accessions KF823816 and KF823817), were identified. In addition, by analysis of the additional obtained sequences of sample 37, the partial viral nucleoprotein (NP) gene (S segment) and partial glycoprotein (G) gene (M segment) of another novel phlebovirus, tentatively called Red fox fecal phlebovirus (NP gene: 606 nt, covered by 33 reads, G gene: 1110 nt, covered by 63 reads, Genbank accessions KF823818 and KF823819) were identified (Figure 4A, B). Also sequences were detected with the closest similarity to sequences of the large segment of phleboviruses, but with a lower coverage of reads. Pairwise identity and phylogenetic analysis of the deduced amino acid sequence of the partial NP and G1 genes with various other viruses of the genus *Phlebovirus* suggest that both viruses are highly divergent viruses belonging to the genus *Phlebovirus* with pairwise identities of less than 34% on the deduced amino acid level of both viruses of both genes (Additional file 1: Table S1, Table S2, Figure 4C, D). Of interest, phleboviruses are transmitted by arthropods or ticks, but these viruses were detected in the fecal material of two animals in this study. It has been demonstrated for Rift valley fever virus that after systemic infection virus can be detected in feces [37], therefore also these animals might have been infected systemically. However, the identification of these viruses in fecal content could be also due to the ingestion of preys (e.g. small mammals) with arthropods or ticks.

### Red fox fecal amdovirus

**Figure 5:**
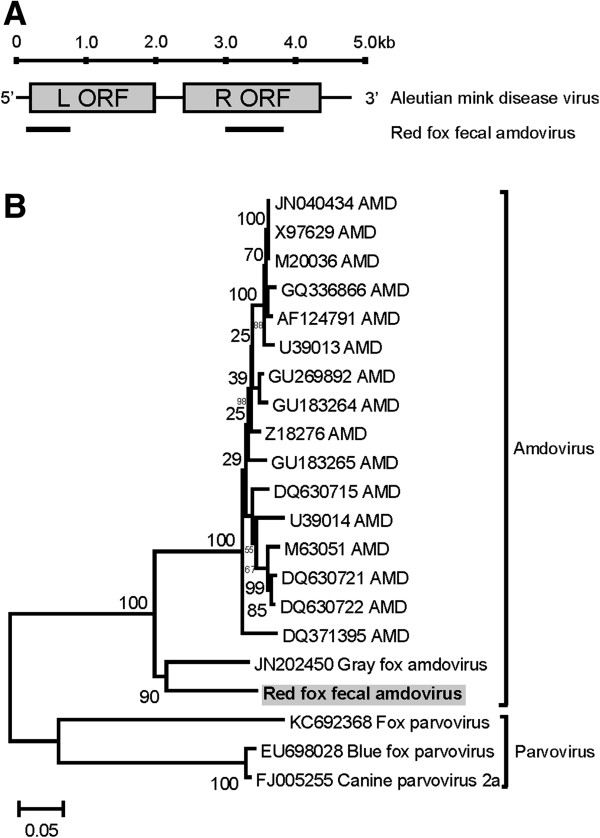
**Phylogenetic analysis of Red fox fecal amdovirus.** Overview of obtained viral sequences of Red fox fecal amdovirus using Aleutian mink disease virus as a reference **(A)**. Phylogenetic neighbor-joining tree with *p*-distance and 1,000 bootstrap replicates of the deduced amino acid sequence of the partial VP2 gene of Red fox fecal amdovirus and VP2 genes of various other viruses of the genus *Amdovirus* detected in mink and gray foxes and viruses of the genus *Parvovirus* detected in red and blue foxes and dogs **(B)**. Indicated are Genbank accession numbers and bootstrap values.

Parvoviruses are small non-enveloped single-stranded DNA viruses. The family *Parvoviridae* has been subdivided into two different subfamilies, *Parvovirinae* and *Densovirinae*. Viruses of the *Densovirinae* infect arthropods, while viruses of the *Parvovirinae* infect vertebrates. At present, the International Committee on Taxonomy of Viruses (ICTV) has recognized eight different genera of the subfamily *Parvovirinae*: *Amdoparvovirus*, *Aveparvovirus*, *Bocaparvovirus*, *Copiparvovirus*, *Dependoparvovirus*, *Erythroparvovirus*, *Protoparvovirus* and *Tetraparvovirus*[22]. The genus *Amdoparvovirus* currently has two members, Aleutian mink disease virus and Gray fox amdovirus. Aleutian mink disease virus causes interstitial pneumonia in young mink and chronic immunological disorders in adult mink, but infection can also occur without clinical signs. Gray fox amdovirus was identified in foxes with abnormal gait and muscle inflammation [38]. In fecal material of red fox sample 40, 11 sequences were detected that had the closest similarity to viruses belonging to the genus *Amdovirus*. Based on these sequences, specific primers were designed and sequences of two partial ORFs (left ORF, putative non-structural gene 1 and 5’ end; 633 nt and right ORF, putative viral protein 2; 830 nt, Genbank accessions KF823809 and KF823808) of this novel virus, tentatively called Red fox fecal amdovirus, were confirmed by Sanger sequencing (Figure 5A). Phylogenetic analysis and calculation of the pairwise identities of the deduced amino acid sequence of the partial VP2 gene revealed that this virus is most closely related to Grey fox amdovirus, with pairwise identities of respectively 83% on the amino acid level and 80% on the nucleotide level (Figure 5B).

### Red fox fecal kobuvirus

Kobuviruses are small single stranded positive sense RNA viruses belonging to the family of *Picornaviridae*. At present, the genus *Kobuvirus* consists of three species, Aichivirus A, B and C. Aichivirus A, B and C were detected in enteric samples from humans, cows and pigs respectively [39-41]. In addition, kobuviruses were detected in fecal samples of a number of other species recently [42]. Although seroprevalence studies suggest that infections with kobuviruses are common, their role as a primary pathogen is unknown [43,44].

**Figure 6:**
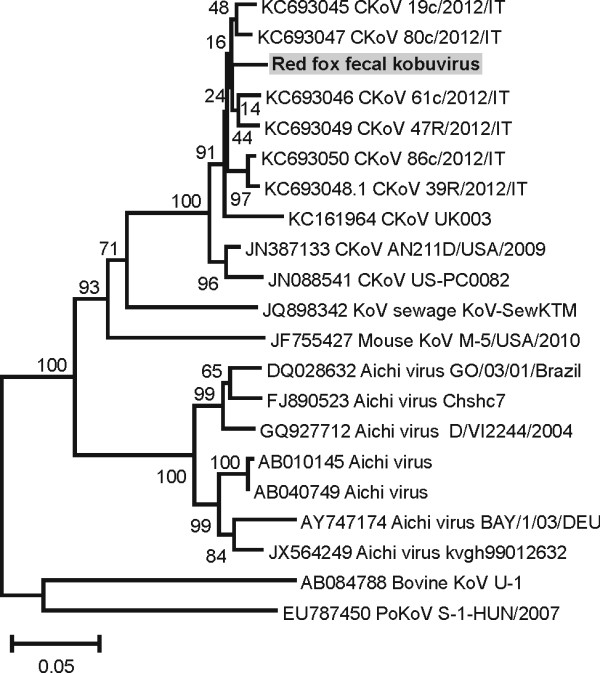
**Phylogenetic analysis of Red fox fecal kobuvirus S37.** Phylogenetic neighbor-joining tree with *p*-distance and 1,000 bootstrap replicates of the nucleotide sequence of the partial 3D gene of Red fox fecal kobuvirus and partial 3D genes of various other viruses of the genus *Kobuvirus*. Indicated are Genbank accession numbers and bootstrap values. CKoV: Canine kobuvirus, KoV: kobuvirus, PoKoV: Porcine kobuvirus.

Sequences with the closest similarity to kobuviruses were detected in a common genet (sample 13) and in two red foxes (samples 37 and 39) (Table 1). Kobuvirus sequences detected in the common genet had the closest similarity to kobuviruses detected in mice and sewage [7,45], while kobuvirus sequences detected in the foxes were nearly identical to kobuviruses detected in healthy and diarrheic dogs [43,46,47]. Using primers described previously [46], the partial 3D region of a variant kobuvirus detected in feces of a red fox (sample 37), tentatively called Red fox fecal kobuvirus (Genbank accession KF823813) was amplified and sequenced. Alignment and phylogenetic analysis of this Red fox fecal kobuvirus with various other kobuviruses indeed confirmed that detected sequences were very similar to canine kobuviruses, with an identity of 97% on the nucleotide and 100% on the deduced amino acid level with Canine kobuviruses detected in Italy recently [46] (Figure 6). The high similarity between kobuviruses detected in dogs and foxes suggest that kobuviruses of dogs and red foxes can be easily transmitted from one host to another or that transmission of these viruses has occurred relatively recently.

### Novel picobirnaviruses

**Figure 7:**
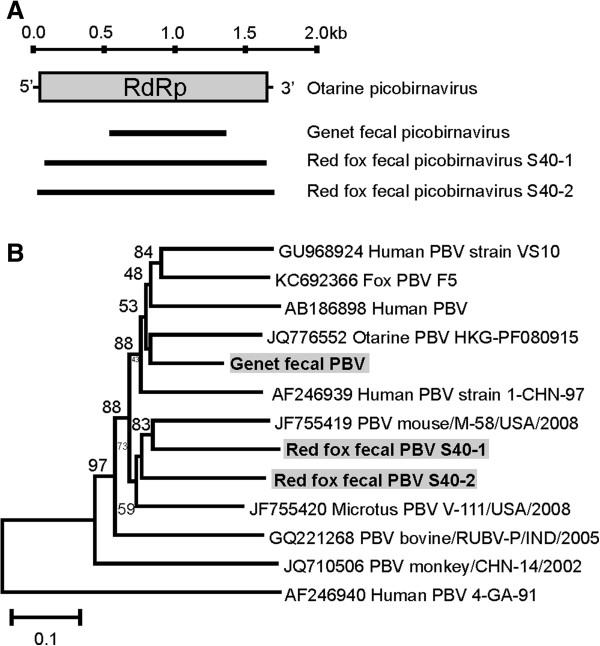
**Phylogenetic analysis of Genet fecal picobirnavirus and Red fox fecal picobirnavirus S40**-**1 and S40-2.** Overview of obtained viral sequences of Genet fecal picobirnavirus and Red fox fecal picobirnavirus S40-1 and S40-2 using segment 2 of Otarine picobirnavirus as a reference **(A)**. Phylogenetic neighbor-joining tree with *p*-distance and 1,000 bootstrap replicates of the deduced amino acid sequence of the RdRp gene of Red fox fecal picobirnavirus S40-1, S40-2 and Genet fecal picobirnavirus and near complete RdRp genes of various other picobirnaviruses. Indicated are Genbank accession numbers and bootstrap values. PBV: picobirnavirus **(B)**.

Picobirnaviruses are small, non-enveloped, bisegmented double-stranded RNA viruses. These viruses have been often detected in fecal samples of humans and various animal species with and without disease [7,14,48,49]. In the present study, sequences that had the closest similarity to viruses belonging to the family *Picobirnaviridae* were detected in fecal samples collected from two common genets, two European minks and two red foxes (Table 1). Based on 454-sequencing reads, the (partial) coding sequences of the RNA-dependent RNA polymerase (RdRp) gene of a novel picobirnavirus detected in one common genet (sample 14; 811 nt, Genet fecal picobirnavirus, Genbank accession KF823812) and of two novel picobirnaviruses detected in a red fox (sample 40, Red fox fecal picobirnavirus 40–1; 1560 nt and 40–2; 1669 nt, Genbank accessions KF823810 and KF823811) were obtained (Figure 7A). Alignment and phylogenetic analysis of these viruses with RdRp gene sequences of similar length of other picobirnaviruses showed that obtained sequences of the RdRp gene were most closely related to picobirnaviruses detected previously in rodents and otarines (Figure 7B).

Various sequences of known and novel viruses were identified but no known zoonotic viruses were detected. In addition to the viral sequences characterized in the present manuscript, a novel rhabdovirus was discovered in feces of a red fox (nr 40, *Bodewes et al*., manuscript submitted for publication), which was divergent from currently known rhabdoviruses, with pairwise identities on the amino acid level of the L gene of this novel rhabdovirus with other viruses of the family *Rhabdoviridae* of only 35% or less.

The presence and detection of the obtained viral sequences is potentially biased by multiple factors, including ecological factors, virus concentrations, a PCR bias, a bioinformatics analysis bias and possible contamination of laboratory kits since we also identified sequences with the closest similarity with NIH-CQV/PHV, although only in one sample [16]. Since for almost all animals only fecal material was available, it is unknown whether these novel viruses have caused disease in the host or if they might potentially transmit to domestic animals or humans. Additional studies based on collection of blood samples and complete necropsies are necessary to elucidate the pathogenicity and epidemiology of these viruses. Of interest, a number of species evaluated in the present study have been the focus of previous viral metagenomics studies in the Netherlands [9,14]. Still, high numbers of previously unknown viruses were detected. This might be due to the relatively low number of animals sampled in each study or due to the geographic distance between the Netherlands and Spain. Further studies using samples of more countries are necessary to elucidate the geographical spread of each of these viruses among animals in Europe and beyond. In addition, in spite of their relevance as potential carriers of pathogens, only a few recent studies have conducted thorough metagenomic analysis of fecal material on wild carnivore species [9,14,50].

## Conclusions

The present study highlights the viral diversity that is present in fecal material of wild carnivores. Results obtained in this study are a step forward in order to improve the limited knowledge about virus diversity present in wild carnivores in Europe and will help to get a better understanding of potential cross-species transmission of viruses between diverse hosts [51] and provide information about potential pathogens [52].

## Materials and methods

### Sample collection

Rectal swabs or fecal specimens were collected from 42 carnivores in the Basque Country and La Rioja regions, Spain (Figure 1; Table 1). Samples from the Canidae, Mustelidae, Viverridae and Felidae families of the order Carnivora, including American mink (n = 10), Common genet (n = 7), Eurasian badger (n = 4), Eurasian otter (n = 1), European mink (n = 9), European pine marten (n = 2); European polecat (n = 3), red fox (n = 4), stone marten (n = 1), and European wild cat (n = 1) were analysed in the present study. Carnivore samples were obtained from found-dead animals, (road casualties and/or poaching) collected by authorized veterinarian personnel of the Wildlife Rehabilitation Centre of Martioda (Alava Regional Council. Department of Environment; Biodiversity section). Additionally, samples from live trapped individuals were obtained in the framework of a systematic population study of the Endangered European mink (*Mustela lutreola*) and the control and eradication program of the invasive alien American mink in Spain, for other purposes than this study (Ref: 3088046-CONSERVISON. Conservation actions for the Endangered European mink: National strategy development, ex-situ conservation program and American mink eradication. TRAGSATEC - Spanish Ministry of Agriculture, Food, and Environment). American minks were humanely killed according to ethical and legal requirements and in agreement with the Spanish law in order to control alien invasive species (Law 42/2007 and Real Decree 1628/2011). All the trapping, handling, sampling and American mink culling was performed by specialized wildlife researchers and veterinarians with the permission of regional wildlife authorities (La Rioja Government. Agriculture, Livestock and Environmental Council. General Direction of Natural Environment. Nature Conservation and Planning Service: Ref: LL/aic; Alava Regional Council. Department of Environment. Biodiversity Section: Ref: 13/32) and in line with the laws and ethical protocols governing wildlife management (Law 42/2007; Decree 14/2002; Foral Order 180/2003 and 322/2003). No animals were sacrificed for the purposes of this study. Therefore, a formal approval by an Institutional Animal Care and Use Committee was not necessary. Following collection, samples were directly stored at −20°C and were stored at −70°C within 2 months after collection until further processing.

### Sequence independent RNA and DNA virus screening of collected samples

Samples were processed for viral metagenomics as described previously [9,49]. In brief, samples were depleted from host nucleic acids and filtered through a 0.45 μM filter. Subsequently, RNA and DNA were extracted using the Nucleospin RNA XS kit (Macherey-Nagel) and the High Pure viral nucleic acids kit (Roche). First and second strand synthesis and random PCR amplification were performed. PCR products were purified and processed for next-generation sequencing with a 454 GS Junior Instrument (Roche). Obtained reads were assembled using *de novo* assembly in CLC Genomics Workbench 5 (CLC Bio) and contigs and individual reads were analyzed by BLASTN and BLASTX respectively. Cut off E-values for significant virus hits for BLASTN and BLASTX were respectively 1.0 × 10^−3^ and 1.0 × 10^−10^. Based on the taxonomic origin of the best-hit sequence, classification of the sequences was performed in MEGAN 4.70.4 [53]. Obtained reads were deposited at the European Nucleotide Archive under archive number PRJEB4910.

### PCR amplification and sequencing

Based on obtained next-generation sequencing data, specific primers of the novel rhabdovirus, theilovirus, and amdovirus were designed to confirm and partially extend the obtained sequence data. Products were sequenced as described previously and primer sequences are available upon request [49].

### Phylogenetic and protein analysis

Nucleotide and/or deduced amino acid sequences of the novel kobuvirus, theilovirus, amdovirus, and picobirnaviruses were aligned using ClustalW in MEGA5 with default parameters [54], while deduced amino acid sequences of the novel phleboviruses were aligned using MAFFT (vs 7; http://mafft.cbrc.jp/alignment/software/) with the E-INS-I algorithm and otherwise default parameters. Phylogenetic analysis of the novel theilovirus, kobuvirus, amdovirus and picobirnaviruses were performed using a neighbor-joining tree with 1000 bootstrap replicates in MEGA5, while phylogenetic analysis of GP and NP genes of the novel phleboviruses was performed using a maximum-likelihood tree (WAG + F + I + G model) with 100 bootstrap replicates. Alignment of the amino acids of the major surface structures of the theiloviruses was performed with JalView version 2 [55]. Prediction of cleavage sites of picornaviral proteases was performed using NetPicoRNA 1.0 (http://www.cbs.dtu.dk/services/NetPicoRNA/). Other conserved motifs were predicted with MEME 4.9.0 (http://meme.nbcr.net/meme/). The presence of conserved domains was evaluated using the Conserved Domains Database [56].

## Competing interests

Two authors of the manuscript have interests to declare: Prof. Dr. A.D.M.E. Osterhaus and Dr. S.L. Smits are part time chief scientific officer and senior scientist respectively of Viroclinics Biosciences B.V. There are no patents, products in development or marketed products to declare. All the other authors have no competing interests.

## Author contributions

RB and ARG designed and performed the experiments, analyzed the data and wrote the manuscript. JMAB and CMES performed the experiments and analyzed the data. ADMEO designed the experiments and analyzed the data. SLS designed the experiments, analyzed the data and wrote the manuscript. All authors read and approved the final manuscript.

## Supplementary Material

Additional file 1: Table S1 Deduced amino acid sequence identities (%) between the partial G gene of Red fox and Otter fecal phlebovirus and selected other viruses belonging to the genus *Phlebovirus*. **Table S2.** Deduced amino acid sequence identities (%) between the partial NP gene of Red fox and Otter fecal phlebovirus and selected other viruses belonging to the genus *Phlebovirus*. **Figure S1.** High divergence of the deduced amino acid sequences of the major capsid loops of various theiloviruses, including Genet fecal theilovirus. Deduced amino acid sequences of the major surface structures of various viruses of the species Theilovirus (VP2 Puffs A and B, VP3 knob and VP1 loops 1 and 2) were aligned. The numbers indicate the locations of the amino acids on the deduced amino acid sequence of the polyprotein of Theiler’s encephalomyelitis virus isolate TOB15 (EU718732). Click here for file
