# Supplementary material for: Viral metagenomic analysis of feces of wild small carnivores
Source: Virol J. 2014 May 15;11:89. doi: 10.1186/1743-422X-11-89 (PMC4030737; doi:10.1186/1743-422X-11-89)

**Additional file 1**

Manuscript

**‘Viral metagenomic analysis of feces of wild small carnivores’**

**Table S2.** Deduced amino acid sequence identities (%) between the partial G gene of Red fox and Otter fecal phlebovirus and selected other viruses belonging to the genus *Phlebovirus*.

|  | **RFFPV** | **OFPV** | **RVFV** | **SFSV** | **CaV** | **ArV** | **ToV** | **BhV** | **LSV** | **HeV** | **SFTX** | **ZTV** | **UuV** | **GAV** | **MaV** | **GoV** |
| --- | --- | --- | --- | --- | --- | --- | --- | --- | --- | --- | --- | --- | --- | --- | --- | --- |
| Red fox fecal phlebovirus |  | 24 | 24 | 21 | 19 | 16 | 24 | 17 | 15 | 20 | 20 | 25 | 25 | 26 | 30 | 23 |
| Otter fecal phlebovirus | 24 |  | 17 | 20 | 22 | 19 | 22 | 22 | 20 | 22 | 20 | 27 | 29 | 26 | 26 | 24 |
| ABD38806 RVFV Smithburn | 24 | 17 |  | 55 | 54 | 48 | 46 | 21 | 21 | 21 | 21 | 23 | 23 | 26 | 25 | 21 |
| AAA75043 Sandfly fever Sicilian virus | 21 | 20 | 55 |  | 56 | 50 | 47 | 18 | 18 | 22 | 23 | 22 | 24 | 23 | 24 | 20 |
| YP_004347992 Candiru virus | 19 | 22 | 54 | 56 |  | 51 | 46 | 23 | 22 | 20 | 22 | 25 | 27 | 26 | 26 | 20 |
| AEB70973 Armero virus | 16 | 19 | 48 | 50 | 51 |  | 42 | 19 | 21 | 22 | 22 | 24 | 25 | 24 | 21 | 20 |
| YP_089671 Toscana virus | 24 | 22 | 46 | 47 | 46 | 42 |  | 21 | 21 | 19 | 20 | 26 | 26 | 25 | 25 | 21 |
| M3811 Bhanja virus | 17 | 22 | 21 | 18 | 23 | 19 | 21 |  | 67 | 26 | 28 | 21 | 21 | 20 | 22 | 20 |
| YP_008003508 Lone star virus | 15 | 20 | 21 | 18 | 22 | 21 | 21 | 67 |  | 25 | 25 | 21 | 21 | 21 | 21 | 16 |
| AFP33393 Heartland virus | 20 | 22 | 21 | 22 | 20 | 22 | 19 | 26 | 25 |  | 70 | 21 | 21 | 21 | 21 | 17 |
| AEO51774 SFTX BX-2010 | 20 | 20 | 21 | 23 | 22 | 22 | 20 | 28 | 25 | 70 |  | 21 | 20 | 21 | 22 | 18 |
| AEL29696 Zaliv Terpenia virus | 25 | 27 | 23 | 22 | 25 | 24 | 26 | 21 | 21 | 21 | 21 |  | 89 | 71 | 52 | 21 |
| AAA79512 Uukuniemi virus | 25 | 29 | 23 | 24 | 27 | 25 | 26 | 21 | 21 | 21 | 20 | 89 |  | 72 | 53 | 23 |
| AFH08733 Grand Arbaud virus | 26 | 26 | 26 | 23 | 26 | 24 | 25 | 20 | 21 | 21 | 21 | 71 | 72 |  | 52 | 21 |
| AFN73043 Manawa virus | 30 | 26 | 25 | 24 | 26 | 21 | 25 | 22 | 21 | 21 | 22 | 52 | 53 | 52 |  | 22 |
| AEJ38174 Gouleako virus | 23 | 24 | 21 | 20 | 20 | 20 | 21 | 20 | 16 | 17 | 18 | 21 | 23 | 21 | 22 |  |

**Table S3.** Deduced amino acid sequence identities (%) between the partial NP gene of Red fox and Otter fecal phlebovirus and selected other viruses belonging to the genus *Phlebovirus*.

|  | **RFFPV** | **OFPV** | **RVFV** | **SFSV** | **CaV** | **ArV** | **ToV** | **BhV** | **LSV** | **HeV** | **SFTX** | **ZTV** | **UuV** | **GAV** | **MaV** | **GoV** |
| --- | --- | --- | --- | --- | --- | --- | --- | --- | --- | --- | --- | --- | --- | --- | --- | --- |
| Fox fecal phlebovirus |  | 27 | 33 | 33 | 27 | 29 | 28 | 33 | 27 | 22 | 28 | 23 | 24 | 26 | 30 | 30 |
| Otter fecal phlebovirus | 27 |  | 26 | 20 | 28 | 29 | 26 | 26 | 24 | 21 | 24 | 30 | 32 | 33 | 28 | 22 |
| ABD38744 RVFV Smithburn | 33 | 26 |  | 59 | 63 | 66 | 62 | 46 | 41 | 41 | 44 | 30 | 32 | 35 | 43 | 28 |
| YP_004382744 Sandfly fever Sicilian virus | 33 | 20 | 59 |  | 60 | 61 | 48 | 40 | 38 | 34 | 37 | 33 | 35 | 35 | 38 | 27 |
| YP_004347995 Candiru virus | 27 | 28 | 63 | 60 |  | 67 | 59 | 44 | 44 | 40 | 43 | 37 | 38 | 35 | 44 | 20 |
| AEB70975 Armero virus | 29 | 29 | 66 | 61 | 67 |  | 54 | 46 | 39 | 39 | 40 | 39 | 41 | 41 | 44 | 26 |
| ACM92017 Toscana virus | 28 | 26 | 62 | 48 | 59 | 54 |  | 41 | 40 | 43 | 40 | 28 | 29 | 29 | 43 | 22 |
| AF066274 Bhanja virus M3811 | 33 | 26 | 46 | 40 | 44 | 46 | 41 |  | 63 | 46 | 52 | 26 | 28 | 27 | 30 | 23 |
| YP_008003509 Lone Star virus | 27 | 24 | 41 | 38 | 44 | 39 | 40 | 63 |  | 54 | 59 | 34 | 35 | 33 | 33 | 22 |
| AFP33391 Heartland virus | 22 | 21 | 41 | 34 | 40 | 39 | 43 | 46 | 54 |  | 65 | 24 | 22 | 23 | 37 | 18 |
| AEO51772 SFTX BX-2010 | 28 | 24 | 44 | 37 | 43 | 40 | 40 | 52 | 59 | 65 |  | 26 | 26 | 27 | 32 | 20 |
| AEL29694 Zaliv Terpenia virus | 23 | 30 | 30 | 33 | 37 | 39 | 28 | 26 | 34 | 24 | 26 |  | 89 | 77 | 45 | 21 |
| AAA47958 Uukuniemie virus | 24 | 32 | 32 | 35 | 38 | 41 | 29 | 28 | 35 | 22 | 26 | 89 |  | 78 | 45 | 23 |
| AFH08734 Grand Arbaud virus | 26 | 33 | 35 | 35 | 35 | 41 | 29 | 27 | 33 | 23 | 27 | 77 | 78 |  | 45 | 24 |
| AFN73044 Manawa virus | 30 | 28 | 43 | 38 | 44 | 44 | 43 | 30 | 33 | 37 | 32 | 45 | 45 | 45 |  | 26 |
| AEJ38173 Gouleako virus | 30 | 22 | 28 | 27 | 20 | 26 | 22 | 23 | 22 | 18 | 20 | 21 | 23 | 24 | 26 |  |

**Figure S1. High divergence of the deduced amino acid sequences of the major capsid loops of various theiloviruses, including Genet fecal theilovirus**. Deduced amino acid sequences of the major surface structures of various viruses of the species Theilovirus (VP2 Puffs A and B, VP3 knob and VP1 loops 1 and 2) were aligned. The numbers indicate the locations of the amino acids on the deduced amino acid sequence of the polyprotein of Theiler's encephalomyelitis virus isolate TOB15 (EU718732).


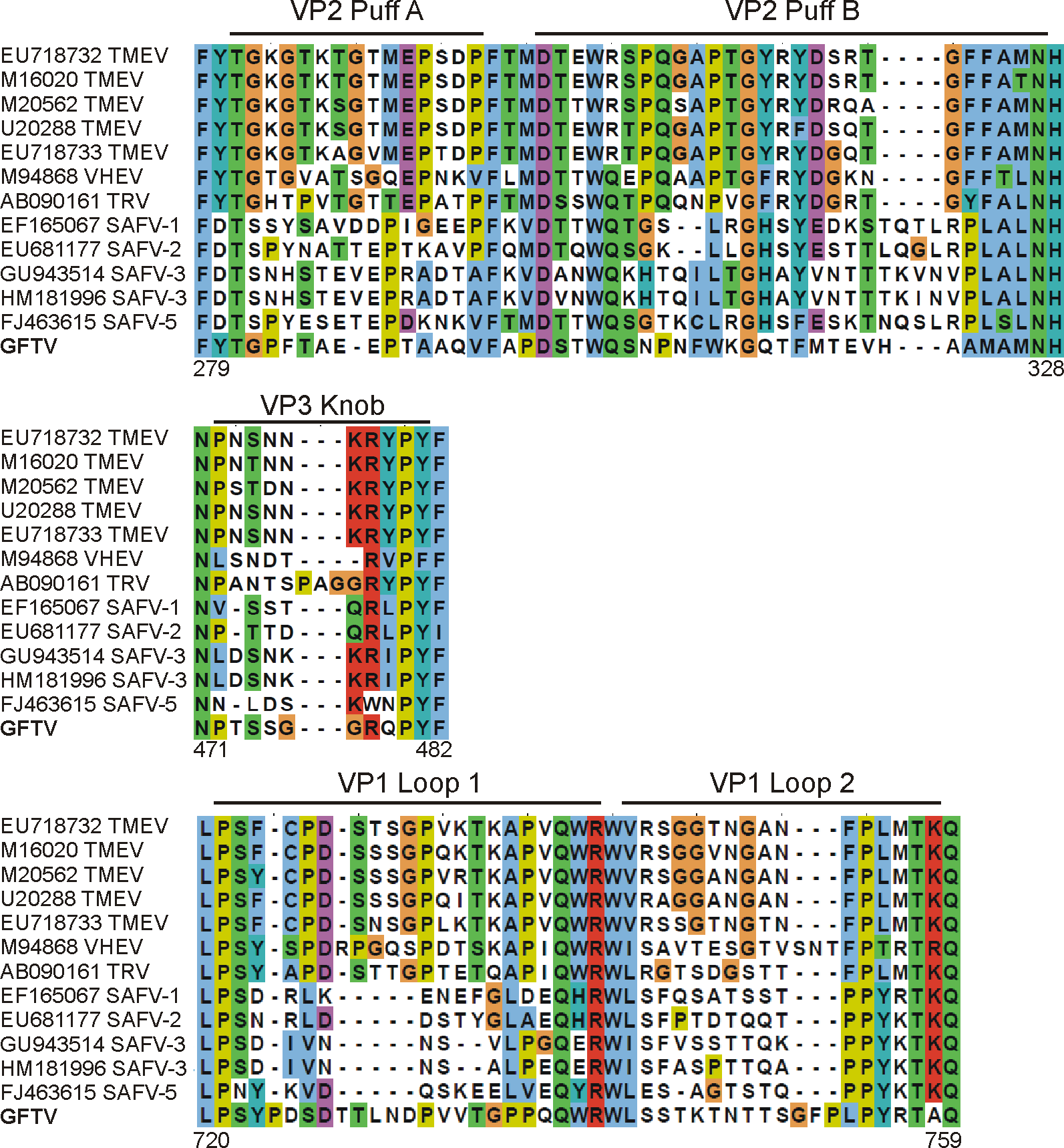

Supplement: Additional file 1: Table S1 — Deduced amino acid sequence identities (%) between the partial G gene of Red fox and Otter fecal phlebovirus and selected other viruses belonging to the genus Phlebovirus. Table S2. Deduced amino acid sequence identities (%) between the partial NP gene of Red fox and Otter fecal phlebovirus and selected other viruses belonging to the genus Phlebovirus. Figure S1. High divergence of the deduced amino acid sequences of the major capsid loops of various theiloviruses, including Genet fecal theilovirus. Deduced amino acid sequences of the major surface structures of various viruses of the species Theilovirus (VP2 Puffs A and B, VP3 knob and VP1 loops 1 and 2) were aligned. The numbers indicate the locations of the amino acids on the deduced amino acid sequence of the polyprotein of Theiler’s encephalomyelitis virus isolate TOB15 (EU718732). [file 1743-422X-11-89-S1.docx]
